# The Features and Treatment Effects on Keratoepitheliopathy for Meibomitis-Related Keratoconjunctivitis

**DOI:** 10.3390/diagnostics14050487

**Published:** 2024-02-23

**Authors:** Yukiko Sonomura, Norihiko Yokoi, Aoi Komuro, Hiroaki Kato, Chie Sotozono

**Affiliations:** Department of Ophthalmology, Kyoto Prefectural University of Medicine, Kyoto 602-8566, Japan; ysonomur@koto.kpu-m.ac.jp (Y.S.); akomuro@koto.kpu-m.ac.jp (A.K.); hiro-kat@koto.kpu-m.ac.jp (H.K.); csotozon@koto.kpu-m.ac.jp (C.S.)

**Keywords:** meibomitis-related keratoconjunctivitis (MRKC), keratoepitheliopathy, clarithromycin

## Abstract

Meibomitis-related keratoconjunctivitis (MRKC) is characterized by meibomitis with corneal epithelial abnormalities, and can be divided into two types: MRKC accompanied with phlyctenular keratitis, and MRKC accompanied with keratoepitheliopathy that is similar to superficial punctate keratopathy (SPK). The purpose of this retrospective study was to investigate the characteristic features of keratoepitheliopathy and treatment outcomes for MRKC. This study involved 27 eyes of 18 MRKC patients (3 males and 15 females). National Eye Institute (NEI) scores and visual acuity were compared at pre and post treatment. All subjects were treated with a small-dose administration of clarithromycin. Keratoepitheliopathy characteristic to MRKC, yet different in appearance from SPK, was noted in 24 of the 27 eyes. Fluorescein staining revealed granular epithelial lesions generally larger than SPK that coexisted with small dark spots. In 17 eyes, keratoepitheliopathy was located within the pupillary zone, and the visual acuity in 12 eyes was less than 1.0. Our findings showed significant improvement in the NEI score in MRKC (*p* < 0.0001) and in visual acuity (*p* = 0.0157) post treatment, and the characteristic features of keratoepitheliopathy in MRKC that are often associated with decreased visual acuity were elucidated. The treatment of clarithromycin was found to be effective for MRKC with keratoepitheliopathy.

## 1. Introduction

The meibomian gland is a type of sebaceous gland within the tarsal plates of the upper and lower eyelids, and it is considered to be a specialized differentiation of the cutaneous sebaceous gland. Meibomian gland orifices emerge just anterior to the mucocutaneous junction on a line posterior to the eyelashes. Moreover, the meibomian glands discharge secretions into ducts, and those secretions, called meibum, mainly contain lipids. Lipids diffuse into the tear film and form the most superficial lipid layer, and are believed to lower the surface tension of the tear film, inhibit evaporation of the tear film, and maintain the optical properties of the cornea by serving as a barrier over the tear film [1]. 

Meibomitis is associated with inflammation within the meibomian glands and is presumably caused by bacteria. In cases of meibomitis, bacterial overgrowth in the meibomian glands is thought to cause chronic inflammation [2]. Moreover, the lid margin can be classified into the anterior region centered on the eyelashes and the posterior region centered on the orifices of the meibomian glands, and blepharitis is accordingly classified as anterior or posterior [3], with meibomitis being included in posterior blepharitis. Meibomian gland dysfunction is classified as a chronic diffuse abnormality of the meibomian glands, and is commonly characterized by terminal duct obstruction and/or quantitative/quantitative changes in the glandular secretion that can result in ocular surface inflammation and alteration of tear film stability, eventually leading to meibomian gland dysfunction with symptoms of eye discomfort. Meibomitis is not synonymous with meibomian gland dysfunction, and meibomian gland dysfunction is one of the possible causes of meibomitis [2]. 

Since meibomian gland orifices are located close to the cornea, meibomitis-associated corneal epithelial damage has been reported in previous studies. Conjunctivitis meibomiana was first described in a study by Elschnig et al. [4], and the findings in that report showed an association between meibomian glands and conjunctivitis or keratoconjunctivitis. In addition, cases of corneal epithelial disorders often accompanied with meibomian gland dysfunction have also been reported [5,6]. In 1977, McCulley and Sciallis [7] found corneal epithelial damage in patients with inflammation and obstruction of the meibomian gland orifices and examined the association with corneal epithelial damage. They reported the obstruction of the meibomian gland orifices on a group of patients with seborrheic dermatitis and rosacea associated with chronic blepharitis, along with corneal epithelial damage, inferring that the break-up time was shorter compared to normal controls and that the corneal epithelial damage was caused by tear film instability due to lipid abnormalities. They termed keratoepitheliopathy and meibomitis as meibomian keratoconjunctivitis. Moreover, Suzuki et al. [8,9,10,11] reported meibomitis associated with phlyctenular keratitis. Originally, it was thought that phlyctenular keratitis was caused by a delayed hypersensitivity reaction to foreign microbial proteins such as *Staphylococcus aureus* and *Mycobacterium tuberculosis*. Suzuki et al. found an association between corneal inflammation and the location of meibomitis, and concluded that phlyctenular keratitis is caused by bacterial proliferation within the meibomian gland [8,9,10,11]. In addition to phlyctenular keratitis, there were cases of superficial punctate keratopathy (SPK) associated with meibomitis, which they termed keratoepitheliopathy with meibomitis as meibomitis-related keratoconjunctivitis (MRKC) in 2000 [12,13,14]. 

MRKC is classified into two types: (1) phlyctenular-type MRKC (PL-MRKC) accompanied with phlyctenular keratitis, and (2) non-phlyctenular-type MRKC (non-PL-MRKC) accompanied with SPK without phlyctenular keratitis [12].

PL-MRKC is diagnosed by characteristic inflammatory nodular-shaped cellular infiltration at the corneal surface with superficial vascular invasion. Since the meibum of PL-MRKC patients is highly positive for *Cutibacterium acnes* (*C. acnes*), and based on the findings of animal-model experiments, researchers have postulated that antigens derived from *C. acnes* may cause a delayed hypersensitivity reaction in the cornea as a pathophysiology of corneal lesions, with a higher prevalence in females and in the presence of specific human leukocyte antigen association [8,9,10]. In addition, it has been reported that oral and intravenous cephem antibiotics eradicate the bacteria in the meibomian glands that cause corneal lesions and are an effective treatment for PL-MRKC [10,11,12].

Non-PL-MRKC is not accompanied with nodular-shaped cell infiltration and manifests keratoepitheliopathy [12,14]. It has been suggested that the pathogenesis of epitheliopathy is characterized by an unstable tear film due to free fatty acids resulting from bacterial lipolysis, evaporative dry eye accompanied with meibomian gland dysfunction, and possible direct epithelial damage due to fatty acids or bacterial exotoxins [12,13,14,15,16,17,18]. For the treatment of non-PL-MRKC, the use of antibiotics such as a cephem antibiotic, minocycline, and clarithromycin has previously been reported [14]; however, a standard treatment protocol has yet to be established. 

In our dry eye outpatient clinic, we found that when treating keratoepitheliopathy with meibomitis, phlyctenular keratitis is characteristic. However, in many cases with corneal epithelial damage, it is often difficult to elucidate the underlying causative disease, and a correct diagnosis generally takes time. Moreover, we have experienced cases of corneal epithelial damage with similar characteristics that responded to oral antibiotic treatment.

In this study, we retrospectively examined the characteristic findings of keratoepitheliopathy that may possibly help in the diagnosis of MRKC, especially non-PL-MRKC, and investigated the efficacy of treatment with low-dose clarithromycin via examining the changes of keratoepitheliopathy and visual acuity between pre and post treatment. 

## 2. Materials and Methods

### 2.1. Subjects 

The protocols of this study were approved by the Institutional Review Board of Kyoto Prefectural University of Medicine (approval no.: ERB-C-1235-3), and were conducted in accordance with the tenets set forth in the Declaration of Helsinki. 

This retrospective study involved 27 eyes of 18 MRKC patients [3 males (*n* = 4 eyes) and 15 females (*n* = 23 eyes); mean age 41.3 ± 18.4 (mean ± SD) years, range: 16–79 years] seen at the Dry Eye Outpatient Clinic at the Kyoto Prefectural University of Medicine Hospital, Kyoto, Japan between June 2010 and February 2017. In all patients, the diagnosis of meibomitis was made based on findings of obstruction of meibomian gland orifices and hyperemia at the lid margin and palpebral conjunctiva by slit-lamp microscopy. 

Patients with obstructive meibomian gland dysfunction, as well as those with obvious inflammation presenting with injection and/or telangiectasia of the conjunctival vessels of the posterior lid margin and tarsal conjunctiva, were included in the study. Cases of anterior blepharitis with inflammation of the eyelid skin and anterior lid margin around the eyelashes were also excluded.

Cases of keratoepitheliopathy accompanied with meibomitis were diagnosed as MRKC. Meibomitis mainly manifesting with nodular cellular infiltration was classified as PL-MRKC, and meibomitis mainly manifesting with corneal epithelial damage-like SPK was classified as non-PL-MRKC.

All subjects diagnosed with MRKC were treated with small doses of oral clarithromycin until improvement of the hyperemia of the lid margin and palpebral conjunctiva as well as the corneal epithelial damage was observed. 

Of the 18 patients in this study, 17 had been referred to our department by their previous physician. Of those, 2 had been diagnosed with suspected MRKC, 2 were noted to have meibomian gland abnormalities such as meibomian gland infarction, chalazion, and hyperemia of the eyelid margin, 1 had been diagnosed with corneal infiltration, and 12 had prolonged corneal epithelial damage and conjunctival hyperemia with no observed improvement. 

Two patients diagnosed with MRKC were referred to us because of difficulties in treatment. Two patients with abnormalities of the meibomian gland did not respond to treatment and were referred to us asking for further examination, while 14 others had been treated mainly for dry eye, but were referred for further examination because of no improvement. 

### 2.2. Ocular Examinations 

Diagnostic examinations were performed from a less invasive procedure. First, the eyelid margin, bulbar conjunctiva, and tear meniscus were examined using a slit-lamp microscope, and then fluorescein staining was performed to confirm tear meniscus abnormality, tear film breakup, and corneal and conjunctival staining. Next, the palpebral conjunctiva and other hidden areas requiring movements were examined via manual contact with the fingers. Fluorescein staining was also performed using a fluorescein test strip (Ayumi Pharmaceutical Corporation, Tokyo, Japan). For fluorescein staining, two drops of saline solution were applied to the test strip and any excess aqueous fluid was then shaken off. The strip was then carefully placed in contact with the center of the eyelid margin to avoid any increase in tear volume. In this study, all cases were evaluated from the medical record and the photographs after fluorescein staining using a slit-lamp microscope.

The characteristic features of corneal epithelial damage were investigated based on the photographs after fluorescein staining, and meibomitis (i.e., the obstruction of meibomian gland orifices and hyperemia at the lid margin and palpebral conjunctiva) and characteristic features of corneal epithelial damage were compared pre and post treatment. Corneal epithelial damage, which is often similar in appearance to SPK, was evaluated using the National Eye Institute (NEI) score (maximum score: 15 points) based on the photographs after fluorescein staining [19]. The cornea was divided into 5 areas, and the corneal staining at each area was scored from 0 to 3 depending on the severity of the staining. The total score of the 5 areas was defined as corneal epithelial damage, and the changes between pre to post treatment were compared. 

Corrected visual acuity was measured at the time of the examination, and a comparison of the visual acuity at pre and post treatment was then made. 

### 2.3. Statistical Analysis

For statistical analysis, the Wilcoxon signed-rank sum test was used for the corneal epithelial staining scores and visual acuity findings. All statistical analyses were performed using R software version 4.1.2. A *p*-value of <0.05 was considered statistically significant.

## 3. Results

**Figure 1:**
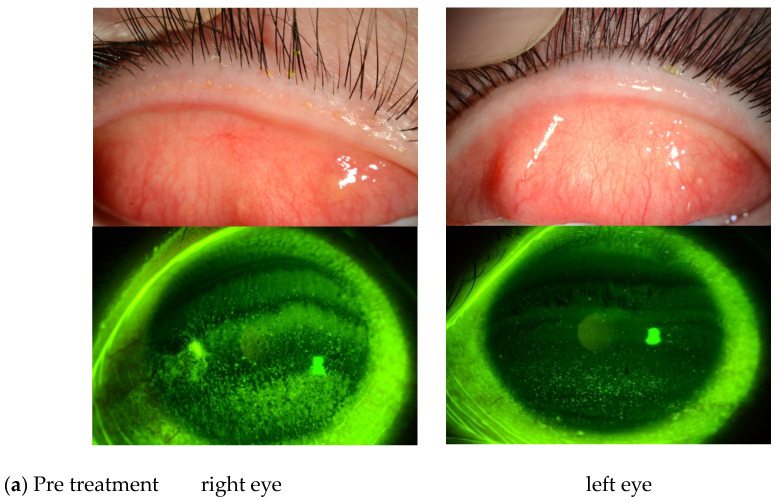

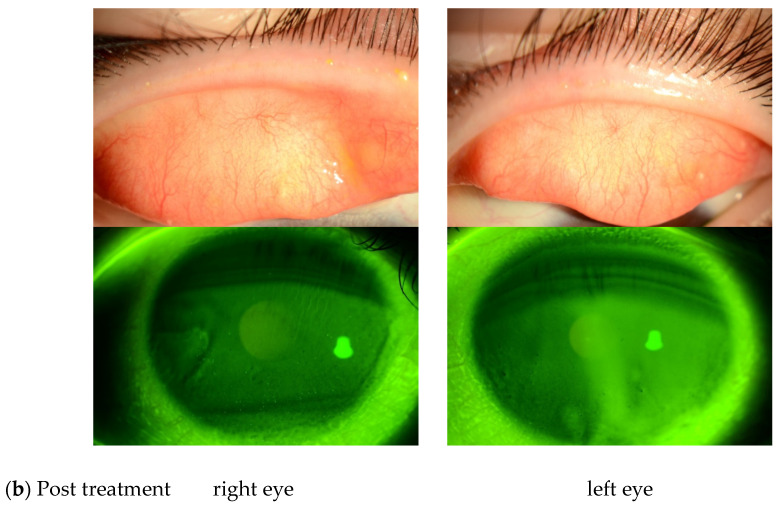
(**a**) Representative case of non-phlyctenular-type meibomitis-related keratoconjunctivitis (non-PL-MRKC) prior to treatment. The diagnosis of meibomitis is made by the hyperemia of the lid margin and palpebral conjunctiva, and by the obstructive meibomian gland function. Fluorescein staining shows granular and slightly larger epithelial lesions than superficial punctate keratopathy (SPK), and the abnormal staining coexisted with small dark spots. (**b**) Representative case of non-PL-MRKC post treatment. Oral clarithromycin at a dose of 200 mg/day and 0.5% erythromycin-based eye drops (4 times daily) were administered. After 3 months of treatment, improvement of hyperemia of the lid margin and tarsal conjunctiva was observed, suggesting improvement of the meibomitis.

Of the 27 eyes in this study, 3 were diagnosed as PL-MRKC and 24 were diagnosed as non-PL-MRKC. Of those 24 non-PL-MRKC eyes, 2 presented with mainly corneal epithelial damage-like SPK and a small nodular-shaped infiltration. The mean age (mean ± SD) of the PL-MRKC patients was 23.5 ± 3.5 years, and that of the non-PL-MRKC patients was 43.6 ± 18.4 years. All subjects were treated with oral administration of clarithromycin in small doses for a duration period that ranged from 2 to 6 months [mean period: 4.4 ± 2.6 (mean ± SD) months]. One pediatric patient was treated with oral clarithromycin at a dose of 50 mg/day, and one patient with severe inflammatory findings was treated with oral clarithromycin at a dose of 400 mg/day during the first week, followed by 200 mg/day thereafter. Sixteen patients were treated with oral clarithromycin at a dose of 200 mg/day. In all but one patient, antibiotic/antimicrobial-based eye drops were used in combination with, and for the same period of time as, the oral administration of clarithromycin, i.e., 0.5% erythromycin-based eye drops (*n* = 16 patients), 0.5% levofloxacin-based eye drops (*n* = 1 patient), and 0.3% gatifloxacin-based eye drops (*n* = 1 patient). In all patients, hyperemia of the lid margin and palpebral conjunctiva and corneal epithelial damage improved after treatment (Figure 1). 

### 3.1. Changes and Features of Corneal Epithelial Damage 

**Figure 2:**
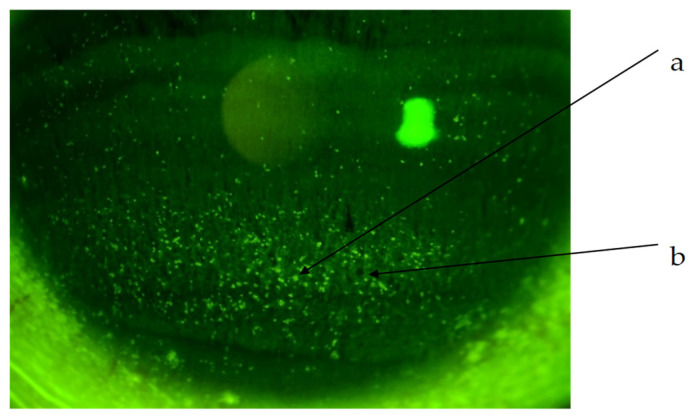
Non-PL-MRKC case with characteristic keratoepitheliopathy. (a) Granular and slightly larger epithelial lesions than SPK. (b) Abnormal staining coexisting with small dark spots.

**Figure 3:**
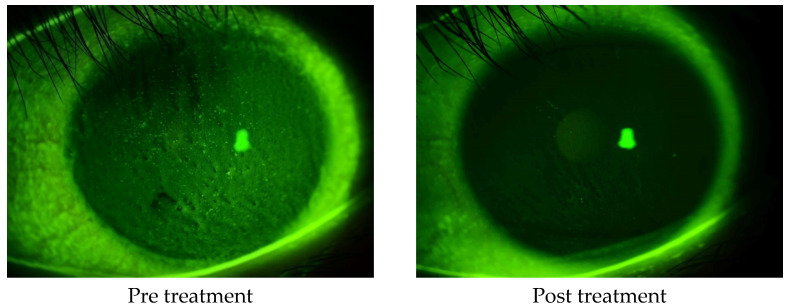
A case of non-PL-MRKC with characteristic keratoepitheliopathy (i.e., granular and slightly larger epithelial lesions than SPK and abnormal staining coexisting with small dark spots) in the pupillary zone. After a 2-month oral administration of clarithromycin at a dose of 200 mg/day and topical administration of 0.5% erythromycin-based eye drops (4 times daily), improvement of hyperemia of the lid margin and tarsal conjunctiva was observed, thus suggesting improvement of the meibomitis. Corrected visual acuity had decreased to 0.5, yet improved to 1.0 after treatment.

**Figure 4:**
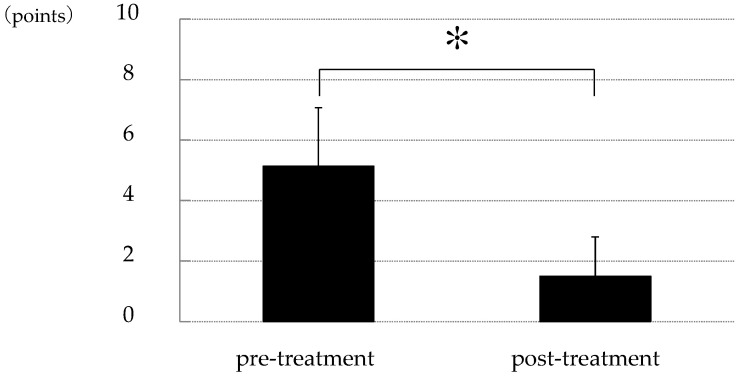
Changes in corneal epithelial damage score between that at pre and post treatment. Compared to that at before treatment, corneal epithelial damage similar to SPK was significantly improved after treatment with oral clarithromycin (*p* < 0.0001) [study of 22 cases; pre-treatment: 5.1 ± 1.9 points; post-treatment: 1.5 ± 1.3 points; based on the National Eye Institute score (a 15-point scale)]. Asterisk indicates a significant difference (*p* < 0.05).

The corneal epithelial damage similar to SPK was found in 24 of the 27 eyes, excluding those with PL-MRKC. Fluorescein staining showed positive results for granular epithelial lesions slightly larger than SPK and small dark spots surrounded by pooled fluorescein (Figure 2). In 17 eyes, the findings extended to the pupillary zone (Figure 3). After treatment, corneal epithelial damage was found to have improved, and the mean corneal epithelial damage score (NEI score) had significantly improved in 22 eyes, excluding 2 eyes. (i.e., pre treatment: 5.1 ± 1.9 points; post treatment: 1.5 ± 1.3 points) (*p* < 0.0001) (Figure 4). Of the 17 eyes with pupillary-region findings, visual acuity had decreased to less than 1.0 in 11 eyes, and 9 of those eyes had characteristic epithelial damage-related decreased visual acuity. 

### 3.2. Changes in Visual Acuity 

**Figure 5:**
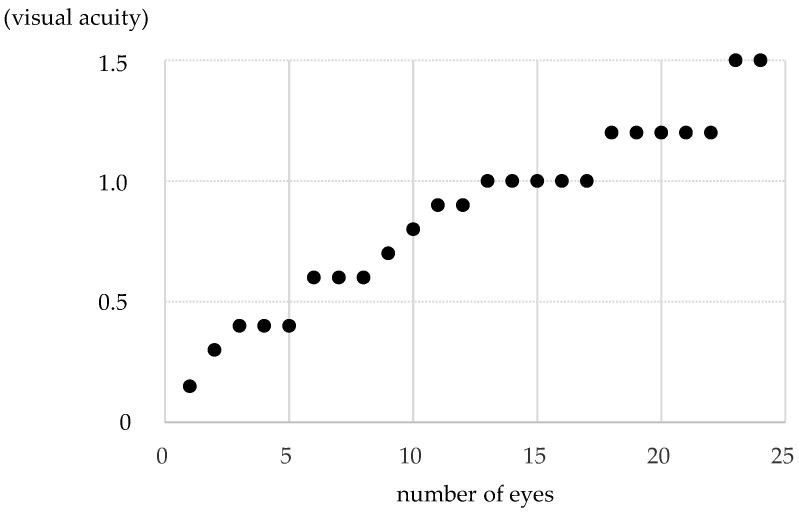
The distribution of pre-treatment corrected visual acuity in non-PL-MRKC eyes. The distribution comprised 0.1–0.5 in 5 eyes, 0.6–0.9 in 7 eyes, and 1.0 or higher in 12 eyes. In 12 of the 24 non-PL-MRKC eyes, the mean corrected visual acuity had decreased to less than 1.0 at prior to treatment.

**Figure 6:**
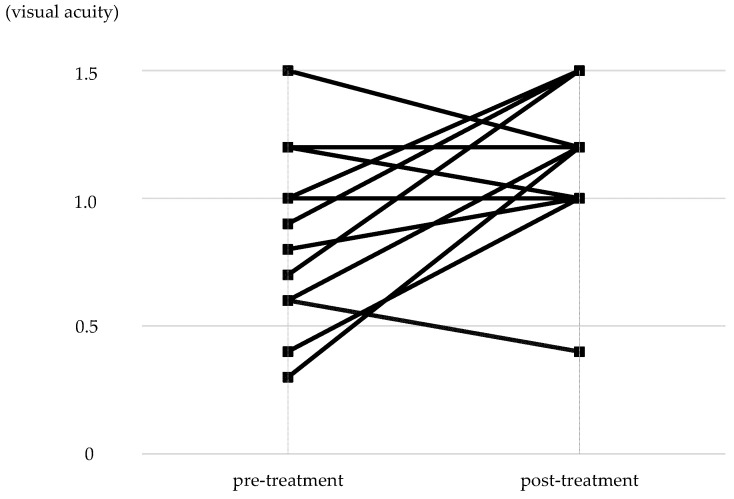
Changes in corrected visual acuity between that at pre and post treatment. In the 17 eyes in which a comparison of corrected visual acuity was possible at pre and post treatment, significant improvement was observed post treatment (*p =* 0.0157).

The distribution of pre-treatment corrected visual acuity in the non-PL-MRKC eyes is shown in Figure 5. The visual acuity distribution comprised 0.1–0.5 in five eyes, 0.6–0.9 in seven eyes, and 1.0 or higher in twelve eyes. In 12 of the 24 non-PL-MRKC eyes, the mean corrected visual acuity had decreased to less than 1.0 (20/20) prior to treatment. In the 17 non-PL-MRKC eyes, in which a comparison of the corrected visual acuity before and after treatment was possible, mean visual acuity had significantly improved after treatment (i.e., pre treatment: 0.8 ± 0.3; post treatment: 1.1 ± 0.3) (*p* = 0.0157) (Figure 6). 

## 4. Discussion

In this retrospective study, we investigated the characteristic features of keratoepitheliopathy that may be useful in the diagnosis of non-PL-MRKC and the efficacy of clarithromycin, a macrolide antibiotic, in the treatment for MRKC. Our findings showed that all non-PL-MRKC eyes had keratoepitheliopathy similar to SPK, which was different from that of common SPK, i.e., granular and generally larger than SPK. Moreover, in those eyes, abnormal staining coexisted with small dark spots surrounded by pooled fluorescein. In addition, a small-dose administration of clarithromycin was found to effectively improve meibomitis and improve corneal lesions in MRKC, and significantly improve characteristic keratoepitheliopathy of non-PL-MRKC.

Meibomitis is a type of posterior blepharitis, and meibomian gland dysfunction is associated with meibomitis. Meibomitis is accompanied by meibomian gland dysfunction, probably due to the inflammation around the orifices of the meibomian glands that lead to their obstruction [2]. McCulley et al. [20] presented a classification of chronic blepharitis, dividing it into two categories, anterior and posterior blepharitis, and also further classified them into six categories. Posterior blepharitis, meibomitis, was classified into primary and secondary meibomitis. Primary meibomitis is associated with acne rosacea and seborrheic dermatitis, and secondary meibomitis is associated with seborrheic blepharitis. It was stated that the primary meibomitis is not primarily an infectious disease, but seems to represent an aspect of generalized sebaceous gland dysfunction. 

Suzuki et al. [12] reported MRKC on meibomitis and associated corneal epithelial disorders, which were thought to be due to bacterial growth without systemic sebaceous gland abnormalities. In that study, meibomitis was described as a type of obstructive meibomian gland dysfunction and severe hyperemia at the palpebral conjunctiva corresponding to the meibomian gland area and at the posterior eyelid margin around the meibomian gland orifices. In this study, as in the above study by Suzuki et al., meibomitis was clinically diagnosed.

Regarding the association between meibomian gland and corneal epithelial damage, some previous studies have described an association between meibomian gland abnormality and conjunctivitis or keratoconjunctivitis, an association between abnormal secretion of meibomian glands and conjunctivitis, and corneal epithelial disorders accompanied with meibomian gland dysfunction [4,5,6]. Previously, McCulley and Sciallis [7] reported that their cases of meibomitis with seborrheic dermatitis, seborrhea sicca, rosacea, or atopic dermatitis presented SPK, and termed keratoepitheliopathy and meibomitis as meibomian keratoconjunctivitis. In a report by Suzuki et al., the authors described cases of phlyctenular keratitis with meibomitis [10,11,12] and cases with meibomitis that manifested SPK without nodular infiltration [12,14]. Their cases were not associated with dermatological rosacea [15], and they termed keratoepitheliopathy with meibomitis as MRKC and their cases with SPK were classified as non-PL-MRKC.

In this study, all subjects with non-PL-MRKC also presented corneal epithelial damage similar to SPK. However, in this present study, fluorescein staining revealed that this finding in non-PL-MRKC was granular and generally larger than SPK, and that abnormal staining coexisted with small dark spots surrounded by pooled fluorescein. Since these findings were seen in all of the non-PL-MRKC eyes, they were considered characteristic abnormal findings that are useful in the diagnosis of MRKC. 

It is noteworthy that corneal epithelial damage extending into the pupillary zone was observed in 17 (70%) of the 24 eyes, and that corrected visual acuity decreased in nine eyes with characteristic keratoepitheliopathy located over the pupillary zone. Suzuki et al. [12] also reported a non-FL MRKC case in which corneal epithelial damage was found on the entire surface of the cornea, resulting in reduced corrected visual acuity. This finding may be in contrast to dry eye. In MRKC, corneal epithelial damage extended into the pupillary zone despite adequate tear volume and decreased visual acuity with characteristic keratoepitheliopathy. In dry eye cases without severe tear deficiency, common non-dense SPK does not seem to decrease visual acuity, even if it is located in the papillary zone. The correlation with keratoepitheliopathy and decreased visual acuity can be useful to differentiate MRKC from other diseases, such as dry eye. As noted above, the characteristic corneal epithelial damage observed by fluorescein staining was granular and generally larger than that in SPK, and that abnormal staining coexisted with small dark spots surrounded by pooled fluorescein. The latter finding had a pattern similar to epithelial edema, and we theorize that the undulations of the epithelium caused the fluorescein to pool around the area, creating a dark spot in the middle. This may have caused the irregular astigmatism and decreased visual acuity, yet further investigation is needed to confirm that speculation. 

McCulley et al. [7] postulated that in their cases, the corneal epithelial damage described as SPK might have been due to tear film instability caused by fatty acid associated with bacterial lipolysis, or direct damage caused by fatty acids, rather than staphylococcal exotoxin [21,22,23]. In addition, since meibomitis is often associated with meibomian gland dysfunction, lipids secreted by the meibomian glands are thought to function in the tear film to retard evaporation from the aqueous layer of the tear film, and it was thought that obstructive meibomian gland dysfunction could result in evaporative dry eye [17,18]. The findings in a study by Suzuki et al. [12] indicated that SPK in non-PL-MRKC might be caused by bacterial exotoxin or damaged by altered lipids [12], and tear film instability caused by fatty acid associated with bacterial lipolysis, or direct damage caused by fatty acids as described by McCulley, and accompanied with evaporative dry eye [14]. As described above, considering that the characteristic corneal epithelial damage seen in this study showed granular and generally larger staining than that in SPK, coexisting with small dark spots surrounded by pooled fluorescein suspected by epithelial edema, and that the epithelial damage was extensive despite the high tear meniscus and inflammation of the conjunctiva but absence of epithelial damage to the bulbar conjunctiva, as in drug toxic keratopathy, we consider that the corneal epithelial damage was caused by bacterial exotoxin rather than evaporative dry eye. The fact that the corneal epithelial damage improved with oral clarithromycin in this study may also support that speculation.

In posterior blepharitis and meibomitis, *Staphylococcus* sp., *Corynebacterium*, and *C. acnes* have been detected. However, they were endogenous bacteria and were not detected consistently, and were not identified as the causative pathogen. It was described that increased free fatty acid suggested bacterial lipolytic enzymes’ involvement [20]. Reportedly, *C. acnes* and *Staphylococcus epidermidis* (*S. epidermidis*) have been detected in meibomian glands, and antigens derived from *C. acnes* have caused delayed allergic reaction in the cornea of PL-MRKC [8,9,10,11]. Similarly, although *C. acnes* and *S. epidermidis* have reportedly been detected in non-PL-MRKC, *S. epidermidis* was more frequently detected in the elderly [14].

In regard to treatment, it has been reported that oral tetracycline was effective with posterior blepharitis and meibomian keratoconjunctivitis [24,25]. Moreover, it reportedly reduces the activity of lipase by bacteria and the production of fatty acids by lipolysis, and this action is suspected to be effective for corneal epithelial damage in meibomian keratoconjunctivitis [26]. Similar effects have been reported with minocycline, a tetracycline antibiotic, which decreased eyelid bacterial flora and decreased diglycerides and free fatty acids for the treatment of posterior blepharitis and meibomitis with acne rosacea or seborrheic blepharitis [27,28]; treatment with oral azithromycin, a macrolide antibiotic, and topical steroid is reportedly effective for the improvement of symptoms with meibomitis [29]. It is thought that *C. acnes* and *chlamydia* are involved in the pathogenesis of phlyctenular keratitis, and it is known that tetracycline, erythromycin, and doxycycline [30,31,32] are effective in the treatment of phlyctenular keratitis. In addition, cephem antibiotic infusion was reportedly beneficial in PL-MRKC [10,11]. Moreover, cephem antibiotic administration and infusion were effective in PL and non-PL-MRKC [12], and oral minocycline, clarithromycin has been proven to be effective for non-PL-MRKC [14]. 

Long-term administration of small-dose 14-membered macrolides is reported to have anti-inflammatory effects for respiratory tract infection diseases involving chronic inflammation [33]. Since this finding was reported, 14-membered ring macrolides have been used effectively for the treatment of other diseases with chronic inflammation and diseases such as chronic rhinosinusitis and acne vulgaris [34,35]. Reportedly, *C. acnes* is highly susceptible to erythromycin, a 14-membered ring macrolide [36]. Clarithromycin, a derivative of erythromycin, has excellent tissue penetration and a long half-life, thus making it suitable for small-dose administration. In this retrospective study, we investigated the efficacy of clarithromycin for the treatment of MRKC in which *C. acnes* is thought to be involved, and our findings showed that it is effective. Moreover, 14-membered ring macrolides are reported to have an anti-inflammatory and an antibacterial effect, as well as immunomodulatory actions that suppress lymphocyte proliferation and the production of inflammatory cytokines. Although 14-membered ring macrolides do not have an antibacterial effect on Pseudomonas aeruginosa, they do suppress pseudomonas exotoxin and reduce bacterial virulence [37,38,39,40,41]. These plural actions probably had an effect on corneal epithelial damage with MRKC. In this present study, there were no side effects in all subjects, so macrolides would possibly be a useful treatment option for non-PL-MRKC.

It should be noted that this present study did have limitations. First, the sample size was small. Second, no bacterial culture of meibum was performed, so the causative organisms could not be identified and the origins of the staining larger than SPK with small dark spots was unclear. Third, cases that manifested small nodular-shaped infiltration and corneal epithelial damage like mixed PL and non-PL-MRKC were observed, and in typical PL-MRKC, similar corneal epithelial damage was also observed. Hence, further investigation involving a larger number of cases is needed. 

Future studies will be needed to confirm the organisms causing the inflammation to determine the detailed morphology of the corneal epithelial damage and its relationship to visual acuity and to determine the mechanism of action of macrolide antibiotics. 

Moreover, non-PL-MRKC eyes had keratoepitheliopathy similar to SPK, which was granular and generally larger than SPK with abnormal staining that coexisted with small dark spots surrounded by pooled fluorescein. A small-dose administration of clarithromycin was found to have effectively improved both meibomitis and keratoepitheliopathy.

It should be noted that only a few of the subjects in this study had been diagnosed with MRKC, and even then, they were referred to us because standard treatment protocol had not been established. Even in cases with abnormalities of the meibomian glands, the relationship with corneal epithelial damage was unclear and did not lead to treatment. Most of the subjects were diagnosed with dry eye or other diseases that did not show any improvement, and treatment was initiated after the first presentation. In the cases in this present study, improvement was observed after oral administration of clarithromycin. 

Previous studies have increased recognition of the association between meibomitis and corneal epithelial damage, and further widespread recognition will lead to diagnosis and treatment in the future. It should also be noted that it is important to confirm the presence or absence of abnormalities at or around the meibomian glands orifices at the eyelid margin after corneal staining to confirm tear film breakup and corneal conjunctival epithelial damage at the time of examination. In addition, when corneal epithelial damage is observed, the characteristic features of corneal epithelial damage identified in this study may help to make each diagnosis more quick and accurate. As for treatment, we found in this study that small-dose long-term macrolide administration was effective without any severe side effect, and that it could be included as an option in the treatment protocol.

In cases of severe and persistent corneal epithelial damage, hyperemia of the lid margin and palpebral conjunctiva is thought to confirm the presence of meibomitis, and whether or not the epithelial damage has the previously described features would help in a differential diagnosis and provide a more effective treatment for non-PL-MRKC.

## 5. Conclusions

In conclusion, the findings in this study elucidated the characteristic features of keratoepitheliopathy in non-PL-MRKC, and show that a long-term small-dose treatment with clarithromycin improves meibomitis and keratoepitheliopathy in MRKC, which may provide better insight into the diagnosis and treatment of MRKC.

## Data Availability

The data presented in this study is available upon request from the corresponding author.

## Abbreviations

*C. acnes*: *Cutibacterium acnes*; MRKC: meibomitis-related keratoconjunctivitis; NEI: National Eye Institute; non-PL-MRKC: non-phlyctenular-type MRKC; PL-MRKC: phlyctenular-type MRKC; *S.* epidermidis: *Staphylococcus epidermidis*; SPK: superficial punctate keratopathy.
